# BOLD and its connection to dopamine release in human striatum: a cross-cohort comparison

**DOI:** 10.1098/rstb.2015.0352

**Published:** 2016-10-05

**Authors:** Terry Lohrenz, Kenneth T. Kishida, P. Read Montague

**Affiliations:** 1 Virginia Tech Carilion Research Institute, Virginia Tech, Roanoke, VA, USA; 2 Department of Physics, Virginia Tech, Blacksburg, VA, USA; 3 Wellcome Trust Centre for Neuroimaging, London, UK

**Keywords:** BOLD signal, dopamine, fast-scan cyclic voltammetry, prediction error

## Abstract

Activity in midbrain dopamine neurons modulates the release of dopamine in terminal structures including the striatum, and controls reward-dependent valuation and choice. This fluctuating release of dopamine is thought to encode reward prediction error (RPE) signals and other value-related information crucial to decision-making, and such models have been used to track prediction error signals in the striatum as encoded by BOLD signals. However, until recently there have been no comparisons of BOLD responses and dopamine responses except for one clear correlation of these two signals in rodents. No such comparisons have been made in humans. Here, we report on the connection between the RPE-related BOLD signal recorded in one group of subjects carrying out an investment task, and the corresponding dopamine signal recorded directly using fast-scan cyclic voltammetry in a separate group of Parkinson's disease patients undergoing DBS surgery while performing the same task. The data display some correspondence between the signal types; however, there is not a one-to-one relationship. Further work is necessary to quantify the relationship between dopamine release, the BOLD signal and the computational models that have guided our understanding of both at the level of the striatum.

This article is part of the themed issue ‘Interpreting BOLD: a dialogue between cognitive and cellular neuroscience’.

## 1. Introduction

The reward prediction error (RPE) hypothesis—that phasic activity in midbrain dopamine neurons reflects a prediction error—has gained wide acceptance [1–4]. Presumably, this prediction error is reflected downstream in fluctuations in dopamine levels in the striatum, which receives dopamine neuron projections. The RPE hypothesis for dopamine has been tested using BOLD responses in human subjects during simple conditioning tasks [5–8]. BOLD activations in the human striatum were consistent with the computational RPE hypothesis. However, findings such as this only established that a slow-to-peak composite signal (BOLD) demonstrated dynamics during learning consistent with the RPE hypothesis. Knutson and co-workers [9] have shown a link between BOLD responses in nucleus accumbens and agonism of postsynaptic dopamine receptors (D1 receptors) suggesting a neural site and type of behavioural paradigms where BOLD responses could act as a proxy for dopamine drive through this structure. In addition, there is evidence that dopamine modulates medium spiny neurons through activation of D1 and D2 receptors [10,11]; findings consistent with dopamine drive and modulation of striatal neurons. With that in mind, there are also reports that dopamine-mediated reward signals decrease BOLD signals in visual cortex [12] where dopamine has also been reported to induce a dissociation between local neural activity and BOLD [13]. On balance, there is not yet a straightforward accounting of how dopamine acts to modulate BOLD. Thus, the precise relationship between the midbrain dopamine neuron spiking, transmitter release in the striatum and the BOLD signal remains unclear. This situation has recently been changed slightly.

First, advances in electrochemistry (fast-scan cyclic voltammetry (FSCV)), for example in rodents [14–17], non-human primates [18–20] and humans [21,22]), when paired with modern inference techniques, have allowed the stable recording of sub-second transients in dopamine. These experiments have confirmed in limited contexts that the spikes in midbrain dopamine neurons representing RPEs translate to corresponding fluctuations in dopamine concentrations [16] (See [23] for one data-driven model of the conversion of spikes to dopamine release).

Secondly, Ferenzci *et al*. [24] have made an important advance in our understanding of the link between dopamine and BOLD. These investigators used optogenetic techniques in rats to establish direct correspondence between (stimulated) spiking midbrain dopamine neurons and measured BOLD signals in striatum. These two advances show that in rodents there is a direct correspondence between spiking midbrain neurons and striatal dopamine (DA) release (from DA measurements in rodent striatum), as well as a direct correspondence between spiking midbrain neurons and BOLD signal in striatum. While the mechanism between DA release and the BOLD signal in striatum is not fully understood, these advances suggest a direct correspondence between dopamine release in striatum and the BOLD signal.

In [22], Kishida *et al*. extended the FSCV technique to humans to measure striatal DA. The participants were Parkinson's patients who underwent surgery for deep brain stimulation (DBS) electrode implantation while playing a sequential investment game. This investment game has previously been coupled with BOLD imaging to investigate neural correlates of computational parameters related to the game [25]. These two datasets present a unique opportunity to make a small step forward in understanding the relationship between BOLD and dopamine release in striatum during a decision-making task.

## 2. Material and methods

For complete details on the BOLD experiment, see [25]; for full details on the FSCV experiment, see [22].

### (a) Participants, BOLD experiment

In total, 54 participants were recruited and research conducted under a protocol approved by the institutional review board at Baylor College of Medicine. The participants provided written consent for the task procedures. The cohort included 31 males and 23 females, aged 19–54.

### (b) Participants, fast-scan cyclic voltammetry experiment

Participants (*n* = 17) provided written consent to a protocol approved by the institutional review boards at Wake Forest University Health Sciences and Virginia Tech. The participants were approached for participation in this study after they were approved as candidates for DBS electrode implantation for treatment of Parkinson's disease. They were informed prior to written consent that if they participated that (i) there would be an additional probe—a carbon-fibre microelectrode, and (ii) the procedure would last up to 30 min longer. The cohort included 16 males and 1 female, aged 42–76.

### (c) Behavioural task, BOLD experiment

**Figure 1.**
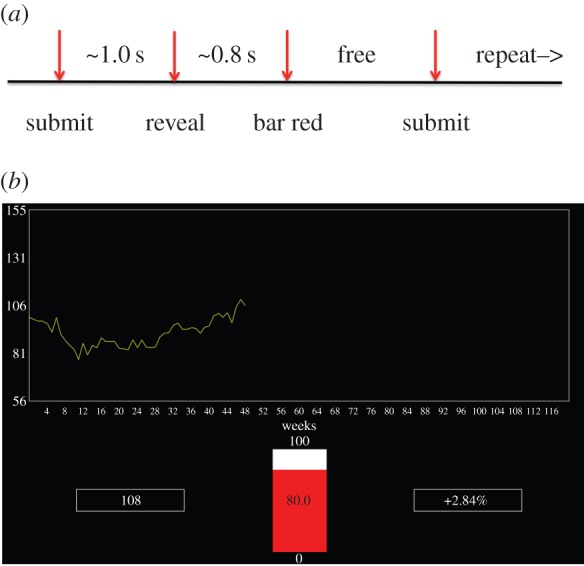
Task design and visual display. (*a*) Timeline of the task. When an investment is submitted the investment bar turns grey. Approximately 1.0 s after submitting, the next snippet of the market is displayed. Approximately 0.8 s after that the investment bar turns red, and the next investment can be submitted. (*b*) Visual display. Figure shows the bar (middle) in the red state (investment can be lodged). On the left is the current portfolio value; on the right is the previous outcome. The market trace is in yellow, and shows the result after two previous investment rounds.

Subjects participated in an investment task in the MRI scanner in which they repeatedly decided what percentage of their assets to risk in ‘markets’ (10 markets in all, 20 decisions per market) represented by traces taken from actual markets. More precisely, after participants were endowed with $100, and saw an initial trace of the market (a total of 10 periods), they used a button box to move a bar on the screen to the percentage of their portfolio in the market desired (0–100% in increments of 10%; see figure 1*a* for timeline). To lodge their decision they pressed one of two buttons on a button box controlled by the other hand. The next segment of the market then appeared (a screen projected onto a mirror in the scanner), and the current portfolio amount and per cent gained or lost was displayed on the screen (figure 1*b*). The process was then repeated for a total of 20 decisions for each market. Subjects also participated in a ‘Not Live’ condition in which 10 additional markets were displayed, but subjects made a visual discrimination. The Not Live markets were alternated with ‘Live’ markets. A total of 200 decisions were made in the Live markets. Participants were paid their final portfolio value in US$.

### (d) Behavioural task, fast-scan cyclic voltammetry experiment

The behavioural task for the FSCV experiment was almost identical to the task for the BOLD experiment. Here, the participants played only six markets (one participant completed only five markets; also, the markets were similar to but not the same as the markets in the BOLD experiment), and also did not participate in the Not Live condition. Additionally, these participants saw a computer screen directly, and were paid based on their final portfolio value. The hand contralateral to the implantation hemisphere manipulated the button box for moving up or down; the hand ipsilateral to the implantation hemisphere submitted the investments using the other button box.

### (e) Procedures, fast-scan cyclic voltammetry experiment

At the beginning of the surgical procedure, as is standard, a Cosman-Roberts-Wells (CRW) stereotactic frame is fastened to the patient's head. Then a volumetric computed tomography (CT) scan is aligned with pre-operative MRI scans. These scans in turn are then aligned to reference scans in the Cranial Vault dataset and atlas [26] using an algorithm on the Wayport Navigator workstation. At this point, the trajectory of the stimulating electrode is selected. The final target of the electrode is either the subthalamic nucleus or the internal segment of the globus pallidus, depending on clinical recommendations; as such, the electrode trajectory may pass through the caudate or the putamen. Before the stimulating electrode is placed, a microelectrode is used to map anatomical boundaries using functional (electrophysiological) properties of the tissue. These recordings and the neuroanatomical images are used to determine the optimal DBS-electrode placement. It is during this stage of the procedure that the carbon-fibre microelectrode is inserted. The recording is taken in the caudate or putamen (see the electronic supplementary material for details). The microelectrode passes through one of five possible microelectrode trajectories defined by the ‘Ben-Gun’ array, and never goes deeper than the microelectrode used for DBS-electrode placement. Once the carbon-fibre microelectrode is in place, a 400 V/s triangular voltage waveform is applied to the electrode (−0.6 V to +1.4 V to −0.6 V in 10 ms), with a 6.67 ms period (potential held at −0.6 V) between applications (60 Hz for the signal), for 10 min. During this time, the patient is reminded about the play of the game and is reinstructed about the use of the handheld button boxes. After the 10 min equilibration protocol, the experimental protocol is started. The same triangular waveform is applied as before, but the wait time between applications is lengthened to 90 ms, so that the actual signal is acquired at 10 Hz.

### (f) Carbon-fibre microelectrode and data acquisition (see [22], for full details)

The carbon-fibre microelectrode was fabricated in-house [21,22]. The carbon-fibre sensor extends approximately 120 µm beyond the polyimide coated fused-silica capillary tubing, which houses a platinum–iridium wire and forms the working electrode. The reference electrode is housed within the microelectrode guide tube, which is identical in construction to the microelectrode guide tubes used for functional mapping during the clinical procedure. The carbon-fibre microelectrode assembly was then connected using shielded cables to a mobile electrochemical recording station, which was comprised of a head stage (CV-7B/EC, Axon Instruments), an amplifier (700B, Axon Instruments), an analogue-to-digital converter (Digidata 1440A, Axon Instruments) and a laptop (MacBook Pro, Apple). The 1440A also collected the button box output, the output of a photodiode on the patient's screen and an additional signal, a square waveform at 1 Hz generated by a Tektronix AFG320 Arbitrary Function Generator, split and sent to the 1440A as well as the behavioural recording system. The current from the electrode was recorded at a frequency of 100 KHz.

### (g) Behavioural recording system

A second laptop (MacBook Pro, Apple) ran in-house software, NEMO, that controlled the behavioural paradigm. The view on the computer screen was exported to a monitor placed in view of the patient. The output of the behavioural stream was synched to the physiological data in two ways: each screen change was accompanied by a small white box on the lower left side of the screen, which was detected by photodiode and relayed to the 1440A; the 1 Hz square wave described above was split and sent to the 1440A and the behavioural recording system.

### (h) Analysis, fast-scan cyclic voltammetry experiment

*Summary*. Our approach is to record *in vitro* the current output of numerous carbon-fibre training electrodes in known, controlled, DA concentrations, and then use cross-validated penalized linear regression to train a model for estimating the *in vivo* DA concentrations from the *in vivo* current recordings.

#### (i) Details, data for model training

**Figure 2.**
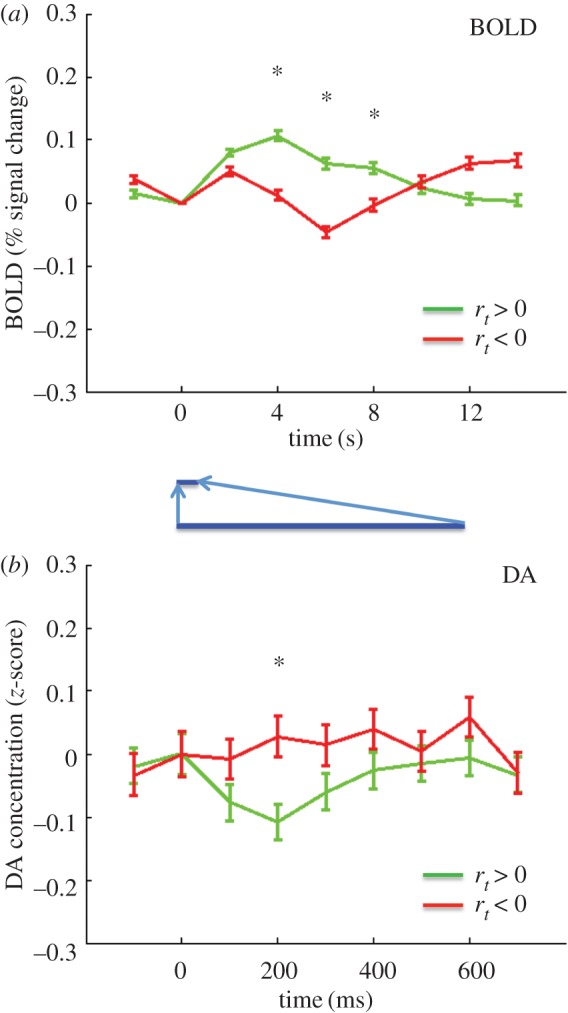
BOLD and DA responses to positive and negative market returns. (*a*) BOLD response (per cent signal change). Green trace, response to positive market return (*N* = 5854); red trace, response to negative market return (*N* = 4946). A star represents difference significant at *p* < .05, FWER corrected (over time points 4, 6 and 8 s – see methods), two-tailed. (*b*) DA response (*z*-score). Green trace, response to positive market return (*N* = 1129); red trace, response to negative return (*N* = 885). A star represents difference significant at *p* < .05, FWER corrected (over time points 200, 300 and 400 ms—see methods), two-tailed. The blue inset highlights the different timescales in the figures.

(1) *Probe selection*. Probe selection is used to identify which calibration datasets will be included in the model-fitting procedure. The ideal calibration dataset would be one that was generated in conditions that exactly match the recording environment and on electrodes that are an exact match in construction and electrochemical properties. This is not possible to achieve in any circumstance as even the exact electrode that was used during surgery undergoes changes during the recording. Empirically, we have found that fitting a model using a single calibration dataset from one electrode to make predictions on another electrode can result in significant error in the resulting predictions. However, if the shape of the voltammogram of the electrode used to generate the calibration dataset is similar to the target probe's voltammogram shape, then the error in the resulting model is reduced substantially. In the near-ideal case, subsampling a calibration dataset for hold-out test samples that do not enter into the model-fitting procedure results in excellent minimal prediction error [22] (figure 2*c*,*d*). In order to decrease the bias—any one electrode may introduce into the resulting concentration prediction model—we train our models using calibration datasets *pooled from multiple electrodes*.

Each calibration set (one from each electrode) contains variations in the voltammogram responses that are characteristic to the controlled changes in dopamine concentration and changes in pH, but also subtle variations due to minor differences in electrode construction (e.g. carbon-fibre length, electrical connections and so on). To determine which of the electrodes from our database of calibration sets we will include, we perform a ‘probe clustering procedure’ to identify which of the calibration datasets best match the gross response profile of the target (or test) electrode. To do this, we use one exemplar voltammogram from each electrode. The exemplar from the patient data is collected from the midpoint of the experiment and the exemplar from the calibration datasets are each taken from a recording in 1× phosphate buffered saline (PBS). The rationale for this is to try to capture the overall voltammogram shape and amplitude that best match the exemplar from the target probe. We then cluster the non-background voltammogram exemplars and choose those that cluster with the target probe's voltammogram as the calibration set that will be used in the model-fitting procedure. *In vitro*, we observe excellent performance in minimizing the concentration prediction error on tests on multiple electrodes not used in the calibration and model-fitting procedure, suggesting we have obtained generalized models for making good estimates of dopamine concentration *in vivo*.

(2) *Data from training probes.* The carbon micro-fibre electrode and reference electrode were placed in a glass-capillary flow cell initially filled with 1× PBS. Powdered dopamine hydrochloride (Sigma-Aldrich) was dissolved in HCl, then further diluted to desired concentrations using 1× PBS. This liquid was then injected while FSCV data were recorded *in vitro* at 100 kHZ using the same voltage sweep used *in vivo.* The data collection sequences consisted of 2 min segments, with the concentration of DA changed in steps during the first 10 s of the segment*.* Data from multiple probes were grouped by subsampling the data according to a normal distributions *N*(*μ*, *σ*) characterized by a concentration mean *μ* with standard deviation *σ*.

*Model training.* The data for training a model consists of an *M x 999* data matrix *x*, and an *M*—vector *y* of DA concentrations from a subset of training data characterized by concentration mean *μ* with standard deviation *σ*. A row *x_ij_*, *j* = 1 to 999, of *X* is the derivative of the current response of a training probe in a DA concentration *y_i_*. The model is a vector *β* (of dimension *N* = 999) which, when, augmented by the constant term, is the solution to a penalized linear regression problem (the elastic net [27])
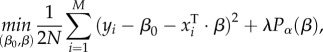
where *P_α_*(*β*) is a term that penalizes the size and number of non-zero elements of
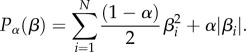
When *α* = 1, this is the lasso [28], and when *α* = 0, this is ridge regression [29]. The question remains how to fix the constants *α* and *λ*. For this, we used cross-validation (cvglmnet in Glmnet [30]). For fixed *α*, cvglmnet calculates a range of *λ* and partitions the data into 10 equal subsets called folds. For each *λ*, the penalized regression problem is solved for 9/10 of the data and used to predict on the remaining 1/10 of the data. The m.s.e. is calculated for this prediction on each fold, and is averaged. The minimum average m.s.e. over the range of *λ*s is recorded. This is repeated over a grid of *α*s from 0 to 1 in 0.1 increments, and the *α*,*λ* pair with minimum average m.s.e. is selected. Finally, with this *α*,*λ* pair the penalized regression problem on all of the training data is solved to obtain the final model (*β*_0_, *β*^T^).

*Model selection for *in vivo* probes.* After the previous training steps, we have a collection of *M* models (index the models by *m* = 1,2, … ,*M*). Recall that a given model *m* is characterized by a training concentration *μ_m_* and concentration range *σ_m_* as a normal distribution. Let the predictions of a model on an *in vivo* dataset be *p_m_*_,*i*_, and define the model error to be
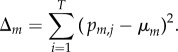


The model used for predicting the *in vivo* DA concentrations is the model with minimum Δ*_m_*

### (i) DA trace processing

DA traces were extracted from 3000 ms before the reveal event, and 3000 ms after. These DA snippets were de-meaned and divided by the standard deviation taken over that snippet. The time zero DA data point was taken as the sample closest in time to the onset of the reveal event.

### (j) DA analysis

The data were pooled across subjects, and for each categorization of the data by behavioural factors (see below in the BOLD methods for definitions of behavioural variables), the DA data were first baseline corrected by subtracting the mean of the DA traces at the reveal time. For plotting purposes, the points 100 ms before and 700 ms after the reveal were used. The traces were then analysed by a two-factor (sign of behavioural variable e.g. MKT, RPE, DBET and time; the DA levels used here were at 200, 300 and 400 ms after the reveal) repeated-measures ANOVA with time as the repeated factor. The analysis was performed in R [31,32] using the function gls in the nlme package [33]. Multiple comparison calculations for significance were performed in R using the function glht in the package multcomp [30].

### (k) BOLD image collection and preprocessing

Images were collected on Siemens Allegra scanners at Baylor College of Medicine. Structural scans (T1) were acquired using an MPRage sequence (Siemens). Functional scans were acquired with the following characteristics: echo-planar imaging, gradient-recalled echo; TR = 2000 ms, TE = 40 ms, flip angle 90°, 64 × 64 matrix, 26 4 mm axial slices yielding 3.4 × 3.4 × 4.0 mm voxels. Preprocessing was performed using standard algorithms in SPM8. A subject's images were first slice-timing corrected. Next, they were motion-corrected by aligning to the first functional scan using a six-parameter rigid body transformation, then unwarped. The mean of the motion corrected was then co-registered to the subject's T1 image. The T1 image was normalized to the Montreal Neurological Institute (MNI) space using unified segmentation and normalization, resampled to 4 × 4 × 4 mm functional voxels, and smoothed with a 8 mm full-width at half-maximum (FWHM) Gaussian kernel.

### (l) Time-series extraction, BOLD experiment

Masks were created in MarsBar [30] centred at MNI coordinates (8,12,4), (−8,8,4), (16,12,−12) and (−16,8,−12), peak activation coordinates with radius 5 mm and sampled into the space of the functional images. These coordinates were the peak-activation coordinates in L/R caudate, L/R ventral striatum/putamen for fictive error and RPE from [25]. A time series was formed for each ROI by averaging the functional images over this mask. Subjects were selected sequentially and assigned an ROI so that the proportions of the four ROIs represented were the same as in the DA subjects (see the electronic supplementary material). Snippets of time series anchored on the Reveal event were then extracted for 10 s prior and 20 s after the event (16pts for 2 s TR) using the interp1 function in Matlab. The 16 point times series were converted to % signal change by subtracting the values by the value at *t* = 0 (time point 6), dividing by the *t* = 0 value, and multiplying by 100.

### (m) Behavioural parameters

The market return at event *i* (*i* = 1 to 200) is 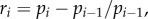 where *p_i_* is the price level on trial *i (p*_0_ is the final price level in the initial price snippet at the beginning of a market). The RPE is defined by
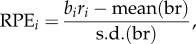
where *b_i_* is the BET at trial *i* and where the mean and standard deviation are taken over the *b_i_r_i_* prior to trial *i* in the current market (for the first return in a market RPE_1_ = *r*_1_; for the second RPE_2_ = *r*_2_ − *r*_1_).

### (n) BOLD analysis

For plotting and analysis*,* the extracted BOLD time series were further reduced to values at nine time points (2 s before reveal, 14 s after). For plotting, the series were binned according to the behavioural variables. For statistical analysis, the data were pooled across subjects and the BOLD values from 4, 6, 8 s were used and entered (in the same manner as for the DA series) into a two-factor repeated measures ANOVA with factors sign of the behavioural variable and time as the repeated factor. The analysis was performed in R [31,32] using the function gls in the nlme package [33]. Multiple comparison corrections for significance were calculated in R using the function glht in the package multcomp [34].

### (o) The NEMO software

The NEMO software is available to others and can be downloaded at http://labs.vtc.vt.edu/hnl/nemo/download.html. The input language for specifying an experiment (stimuli, timing, etc.) is jython and so is flexible, but not particularly point-and-click. NEMO is excellent for arbitrating multiple-subject, multiple-site experiments and streaming the data to a database.

## 3. Results

**Figure 3.**
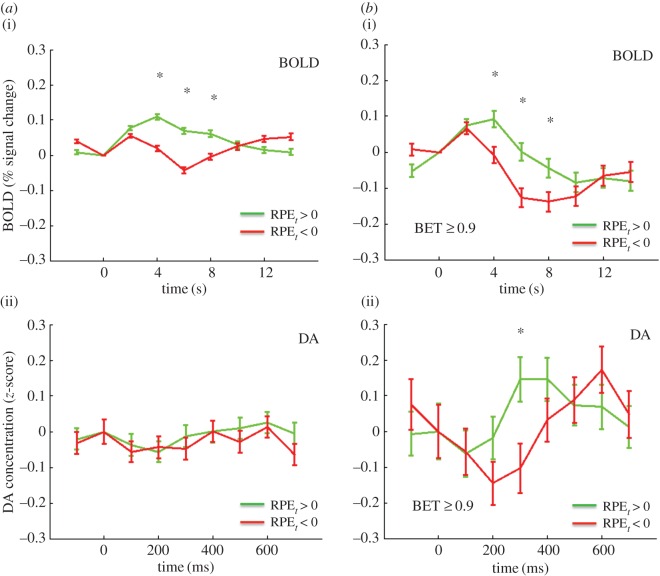
(*a*) BOLD and DA responses to reward prediction errors (RPEs). (i) BOLD response to positive (green trace, *N* = 5143) and negative (red trace, *N* = 5591) RPEs. A star represents difference significant at *p* < .05, FWER corrected, two-tailed. (ii) DA response to positive (green trace, *N* = 1018) and negative (red trace, *N* = 991) RPEs. Differences *p* > .05, FWER corrected, two-tailed. (*b*) BOLD and DA responses RPEs with BET greater than or equal to 0.9. (i) Bold response to positive (green trace, *N* = 675) and negative (red trace, *N* = 679) RPEs and BET greater than or equal to 0.9. A star represents difference significant at *p* < .05, FWER corrected, two-tailed. (ii) DA response to positive (green trace, *N* = 238) and negative (red trace, *N* = 216) RPEs with BET greater than or equal to 0.9. A star represents difference significant at *p* < .05, FWER corrected, two-tailed.

**Figure 4.**
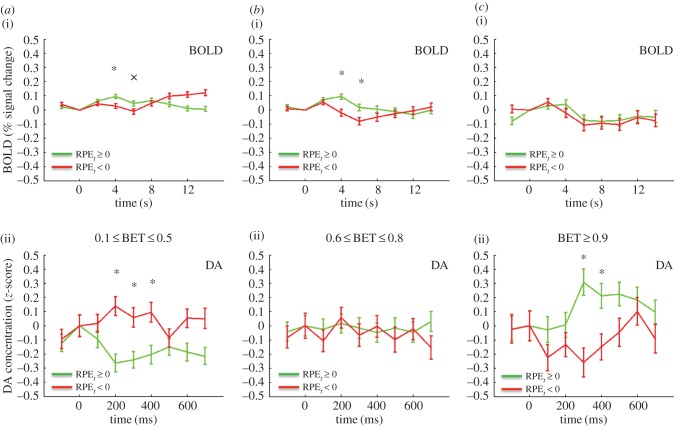
BOLD and DA responses to reward prediction errors (RPEs; absolute value of RPEs less than or equal to 0.75) for varying BET ranges. (*a* (i)) BOLD response to positive or zero (green trace, *N* = 1375) and negative (red trace, *N* = 1125) RPEs and BET range 0.1–0.5 (only events with RPE and market price change the same considered). A star represents difference significant at *p* < .05, FWER corrected, two-tailed; cross represents difference trend-level significant at *p* < .1, FWER corrected, two-tailed. (ii) DA response to positive (green trace, *N* = 246) and negative (red trace, *N* = 194) RPEs and BET range 0.1–0.5. A star represents difference significant at *p* < .05, FWER corrected, two-tailed. (*b* (i)) BOLD response to positive or zero (green trace, *N* = 702) and negative (red trace, *N* = 606) RPEs and BET range 0.6–0.8. A star represents difference significant at *p* < .05, FWER corrected, two-tailed. (ii) DA response to positive (green trace, *N* = 194) and negative (red trace, *N* = 149) RPEs and BET range 0.6–0.8. Differences *p* > .05, FWER corrected, two-tailed. (*c*(i)) BOLD response to positive or zero (green trace, *N* = 329) and negative (red trace, *N* = 280) RPEs and BET range 0.9–1. Differences *p* > .05, FWER corrected, two-tailed. (ii) DA response to positive (green trace, *N* = 119) and negative (red trace, *N* = 99) RPEs and BET range 0.9–1. A star represents difference significant at *p* < .05, FWER corrected, two-tailed.

We first sought to compare the BOLD signal and the DA signal for positive and negative market returns, defined as the percentage return *r_t_* of the market at trial *t*. Figure 2*a* shows the BOLD response in the right caudate for *r_t_* > 0, green, *r_t_* < 0, red. The time series extends from 2 s before to 14 s after the reveal of the market return. Figure 2*b* shows the DA response, again *r_t_* > 0, green, *r_t_* < 0, red. This time series extends from 100 ms before the reveal, to 700 ms after the reveal. The BOLD signals separate in a way consistent with considering *r_t_* as a value signal: the *r*_*t*_ > 0 trace lies above the *r*_*t*_ < 0 trace (difference significant at *p* < .05, FWER, two-tailed, for time points 4, 6, 8 s). For the DA the picture is reversed: the *r*_*t*_ > 0 trace lies below the *r*_*t*_ < 0 trace (difference significant at *p* < 0.05, FWER corrected, two-tailed, for the time point 300 ms). While this first result for *r_t_* is striking, it may be that while *r_t_* is an important computational variable, it may not be the ‘correct’ one here. Thus, we investigated the BOLD and DA signals for the RPEs. The RPE was defined (see Material and methods above) as the current *z*-score of the subject's return *br* (‘current’ meaning the *z*-score over all of the returns in that particular market up to time *t*). Figure 3*a*(i) shows the BOLD response for RPE > 0 green, and RPE < 0, red. Figure 3*a*(ii) shows the corresponding DA RPE > 0 green, and RPE < 0, red. The result is in part similar to that of *r_t_*: the BOLD signal separates, but unlike for market return, the DA signal does not (BOLD: difference significant at *p* < .05, FWER corrected, two-tailed for 4, 6, 8 s; DA, *p* > .05, FWER corrected, two-tailed). However, recalling Kishida *et al.* [22], for DA the RPE fluctuations depend on the size of the investment. Specifically, Kishida *et al.* [22] showed that for large investments the DA signal did separate according to the sign of the RPE. Indeed, for investments greater than or equal to 0.9 both the BOLD and DA signals separate: figure 3b(i) BOLD, 3b(ii) DA (BOLD: difference significant at *p* < 0.05, FWER corrected, two-tailed, time points 4, 6 and 8 s; DA: difference significant at *p* < .05, FWER corrected, two-tailed, for time point 300 ms). Kishida *et al*. [22] went further and systematically investigated the influence of investment size on the relative behaviour of the DA time series with respect to the sign of the RPE. Figure 4 compares the DA signal with the BOLD response in this situation. As in Kishida *et al.* [22] we restrict to |RPE ≤ 0.75|. In figure 4*a*, (i) is the BOLD response for BETS 0.1–0.5 (for these small BETS as in [22] we further restrict to events where the RPE and the market price change are the same sign). The response separates at 4 and 6 s, with the positive RPEs trace over the negative RPEs trace, (difference significant at *p* < .05, FWER corrected, two-tailed, for time point 4 s; difference trend-level significant at *p* < .1, FWER corrected, two-tailed, for time point 6 s). Recapitulating [22] the DA response, figure 4*a*(ii) is inverted with the negative RPE trace over the positive RPE trace (difference significant at *p* < .05, FWER corrected, two-tailed, for time points 200, 300, and 400 ms). Figure 4*b* shows the situation for BETS 0.6–0.8. Figure 4*b*(i) shows that again the BOLD signal separates at 4 and 6 s, with positive RPEs over negative RPEs (difference significant at *p* < .05, FWER corrected, two-tailed, for time points 4 and 6 s), but figure 4*b*(ii) shows the DA signal does not (difference *p* > .05, FWER corrected, two tailed). Finally, figure 4*c* examines the case of BETS 0.9–1. Here, the BOLD does not separate (figure 4*c*(i); difference *p* > 0.05, FWER corrected, two-tailed), but strikingly the DA trace does, and is inverted from figure 4*a*(ii) with the positive RPE trace now above the negative RPE trace (figure 4*c*(ii); difference significant at *p* < .05, FWER corrected, two-tailed, for time points 200 and 300 ms. The inversion of the DA signal for different BET sizes was interpreted in [22] as a counterfactual signal modulating the RPE.

## 4. Discussion

Here, we have used a unique set of data, BOLD data from [25] and FSCV data from [22] to examine the relationship between BOLD and FSCV signals in human striatum sorted by values of computational learning parameters. Previous research using BOLD in humans has reported signals in striatum corresponding to prediction errors incorporated by computational learning models [6–8]. Work in rodents has identified phasic dopamine fluctuations as encoding RPEs [16]. Experiments in non-human primates and rodents show that midbrain dopamine neurons encode RPEs in spike rates [2–5]. Very recent work has shown a relationship in rats between midbrain DA neuron spiking and BOLD signals in the striatum [19]. Altogether, this tempts one to expect to find a tight triad amongst spike rates in DA neurons in the midbrain, DA release in the striatum, and BOLD signals in the striatum. Yet other evidence suggests that the triad is not that tight. Fenrenzci *et al*. [24] also show that optogenetic stimulation of the mPFC in rats dampens the striatal BOLD response to optogenetic stimulation of midbrain DA neurons. Additionally, they show that administering dopamine agonists attenuates the BOLD signal. Taken together this shows that the BOLD response in the striatum cannot be due simply to the dopamine release. It is most probably a complex interaction of dopamine release, binding of dopamine at postsynaptic dopamine receptors, synaptic input from modulatory brain regions, and spiking of striatal neurons, such as medium spiny neurons [10].

In this work, we have exhibited multiple situations where there is no simple correspondence between the BOLD signal and DA measured by FSCV. One immediate possibility is that we are getting unexpected results in the dopamine subjects because these are patients with Parkinson's disease, a disease of the dopamine system. This is possible, but the Parkinson's patients are able to make decisions (financial, consent) that require a functioning dopamine system. Further, we compared using a simple linear regression the decision-making patterns of the Parkinson's patients with the healthy controls. There were no significant differences between the groups (see the electronic supplementary material). Perhaps more interesting is figure 4, which shows the DA signal inverting as investment size goes from smaller to larger, but no such inversion for the BOLD signal. The interpretation of this signal proposed in [22] is that the dopamine signal encodes a linear combination of a prediction error signal and a counterfactual error signal. This is intriguing in light of the fact that it is known that there is heterogeneity in the type and projections of dopamine neurons in the midbrain (with respect to responses to reward and aversive events) [35,36]. This heterogeneity could perhaps help explain how the dopamine transients encode a composite error signal. However, as the BOLD does not invert with BET increases, it is clear in this situation that there is not a simple one-to-one correspondence of DA with BOLD and that a more complicated process is in play.

## Supplementary Material

Additional Information

## Data accessibility

The data from this paper (BOLD and FSCV) will be deposited in the Dryad depository.
