# Supplementary material for: BOLD and its connection to dopamine release in human striatum: a cross-cohort comparison
Source: Philos Trans R Soc Lond B Biol Sci. 2016 Oct 5;371(1705):20150352. doi: 10.1098/rstb.2015.0352 (PMC5003854; doi:10.1098/rstb.2015.0352)
Supplement: Additional Information [file rstb20150352supp1.pdf]

BOLD and its connection to dopamine release in human striatum: a cross-cohort comparison
Terry Lohrenz, Kenneth T. Kishida and P. Read Montague
Phil. Trans. R. Soc. B doi: 10.1098/rstb.2015.0352

Supplementary material

1. Carbon-fiber electrode recording targets.

Left Caudate: 10 patients
Right Caudate: 4 patients
Left Putamen: 2 patients
Right Putamen: 1 patient.

2. Comparison of behavior between healthy controls and Parkinson's patients.

We performed a linear mixed-model regression on the behavioral data for the healthy controls and the Parkinson's patients. The subject's bet was regressed on the previous bet and the most recent market return ($MKT_t = \frac{P_t - P_{t-1}}{P_{t-1}}$). The healthy controls were the "base case" and the Parkinson's patients were coded with a dummy variable GROUP. Below is the output of the *R* function [1] *lme* in the package *nlm* [2].

```
Linear mixed-effects model fit by REML
 Data: behav
  Subset: BETNUM < 20
        AIC       BIC   logLik
  -3455.812 -3361.348 1740.906

Random effects:
 Formula: ~+BET + MKT | SUBJ2
 Structure: General positive-definite, Log-Cholesky parametrization
            StdDev    Corr
(Intercept) 0.1021052 (Intr) BET
BET         0.1879858 -0.765
MKT         0.9880122  0.219 -0.450
Residual    0.2004560

Fixed effects: NEXTBET ~ as.factor(GROUP) * BET + as.factor(GROUP) * MKT
                            Value Std.Error    DF   t-value p-value
(Intercept)             0.1709489 0.0146822 10508 11.643259  0.0000
as.factor(GROUP)2       0.0102323 0.0424430    69  0.241083  0.8102
BET                     0.5873384 0.0272339 10508 21.566406  0.0000
MKT                     1.1319688 0.1379977 10508  8.202806  0.0000
as.factor(GROUP)2:BET   0.0604198 0.0731728 10508  0.825714  0.4090
as.factor(GROUP)2:MKT  -0.1138062 0.3841981 10508 -0.296218  0.7671
 Correlation:
                       (Intr) as.(GROUP)2 BET    MKT    a.(GROUP)2:B
as.factor(GROUP)2      -0.346
BET                    -0.773  0.267
MKT                     0.199 -0.069      -0.412
as.factor(GROUP)2:BET   0.288 -0.831      -0.372  0.153
as.factor(GROUP)2:MKT  -0.072  0.109       0.148 -0.359 -0.238

Standardized Within-Group Residuals:
         Min           Q1          Med           Q3          Max
-4.944352000 -0.521361134  0.009255374  0.483002483  5.447986106

Number of Observations: 10583
Number of Groups: 71
```

Focusing on the fixed effects, the terms which code the difference between the two groups are not significant (as.factor(GROUP)2, as.factor(GROUP)2:BET, as.factor(GROUP)2:MKT; see below for table of fixed effect results).

The model can be written as:

$$BET_{i,j} = \beta_0 + 1_{PD} + (\beta_1 + \beta_2 \cdot 1_{PD}) \cdot BET_{i,j} + (\beta_1 + \beta_2 \cdot 1_{PD}) \cdot MKT_{i,j} + \varepsilon_{1,i} + \varepsilon_{2,i} \cdot BET_{i,j} + \varepsilon_{3,i} \cdot MKT_{i,j} + \varepsilon_{i,j}$$

where $i = 1, 2, 3, \ldots, 71$ subjects, and j is the index for trial. 1_{PD} is 1 if the subject i is a PD patient, 0 otherwise. $\varepsilon_i = (\varepsilon_{i,1},, \varepsilon_{i,2},, \varepsilon_{i,3})^T \sim N(0, \Sigma)$ are the random effects indexed by subject. $\varepsilon_{i,j}$ is the observation error term which is assumed to be IID normal (IID across subjects and observations). The random effects are assumed to be independent of the error term, and the random effects are assumed to be independent across subjects.

Table of Fixed Effects Regression Results

**Predictor**	Estimate	Standard Error	t-value	p-value (two-sided)
1	0.1709	0.1468	11.6433	0.0000
I_PD	0.0102	0.0424	.2411	0.8102
BET	.5873	0.0272	21.5664	0.0000
1_PD*BET	0.0604	0.0732	0.8357	0.4090
MKT	1.1320	0.1380	8.2028	0.0000
1_PD*MKT	-.1138	0.3842	-.2962	0.7671

References.

1. Team, R. C. 2015 *R: A Language and Environment for Statistical Computing*. Vienna, Austria: R Foundation for Statistical Computing.

2. Pinheiro, J., Bates, D., DebRoy, S., Sarkar, D. & Team, R. C. 2015 *nlme: Linear and Nonlinear Mixed Effects Models*
